# Vascular Shunts in Civilian Trauma

**DOI:** 10.3389/fsurg.2017.00039

**Published:** 2017-07-20

**Authors:** Adham N. Abou Ali, Karim M. Salem, Louis H. Alarcon, Graciela Bauza, Emmanuel Pikoulis, Rabih A. Chaer, Efthymios D. Avgerinos

**Affiliations:** ^1^Division of Vascular Surgery, University of Pittsburgh Medical Center, Pittsburgh, PA, United States; ^2^Division of Trauma and General Surgery, University of Pittsburgh Medical Center, Pittsburgh, PA, United States; ^3^Uniformed Services University of Health Sciences, Bethesda, MD, United States

**Keywords:** vascular injury, vascular trauma, vascular shunts, vascular surgical procedures, civilian trauma, military trauma, extremity trauma

## Abstract

Experience with temporary intravascular shunts (TIVS) for vessel injury comes from the military sector and while the indications might be clear in geographically isolated and under resourced war zones, this may be an uncommon scenario in civilian trauma. Data supporting TIVS use in civilian trauma have been extrapolated from the military literature where it demonstrated improved life and limb salvage. Few non-comparative studies from the civilian literature have also revealed similar favorable outcomes. Still, TIVS placement in civilian vascular injuries is uncommon and by some debatable given the absence of clear indications for placement, the potential for TIVS-related complications, the widespread resources for immediate and definitive vascular repair, and the need for curtailing costs and optimizing resources. This article reviews the current evidence and the role of TIVS in contemporary civilian trauma management.

## Introduction

Each year approximately 41 million emergency department visits and 2.3 million hospital admissions are the result of trauma in the United States (1). Extremity vascular injury occurs at a rate of approximately 0.5–4% of trauma admissions (1). Vascular trauma may occur as a result of iatrogenic, penetrating, or blunt injuries to the extremity; 80% though are secondary to penetrating trauma (1). Historically, Debakey and Simeone calculated the amputation rate of vascular injuries during World War II to be greater than 40% (2). Advancement in medical care reduced this amputation rate to approximately 8% during the Vietnam wars (3). A current analysis of the National Trauma Databank (NTDB) showed that current civilian amputation rates for upper and lower extremity arterial injuries are 1.3 and 7.8%, respectively (4).

Current treatment strategies of civilian vascular trauma arise from wartime observations. Rapid diagnosis, hemorrhage control, resuscitation, and operative intervention remain the mainstays of treatment. Vascular structures that are not amenable to ligation or embolization often require exposure and reconstruction, however, critically ill patients may not survive the physiologic stress of definitive revascularization. French surgeon Professor Tuffier first described the clinical use of paraffin-coated silver tubes as temporary intravascular shunts (TIVS) in 1915. Intravascular shunts and techniques evolved over the twentieth century until contemporary shunting techniques for arterial injury were described in 1971 (5).

Military literature has demonstrated the use of TIVS techniques as a bridge to definitive management, primarily since wartime injuries may happen in remote locations where extended travel may be required before centers that provide definitive management can be reached. Controversy exists over the use of TIVS in civilian trauma due to the widespread availability of level I trauma centers in the US where definitive management is possible; however, there may be specific patient populations or injury patterns in which TIVS may provide benefit. While liberal use of shunting for critical illness or distal perfusion during complex and prolonged reconstruction may provide benefit, these techniques are not without complications.

This article reviews the current evidence and the role of TIVS in contemporary civilian trauma management.

## Indications for Shunt Placement

A review of the NTDB showed that the use of TIVS among civilians was most common in blunt trauma (251 of 395 shunted patients: 64%) mostly from motor vehicle collisions with concurrent orthopedic and soft tissue injuries occurring in 185 of the 251 patients (74%). Gunshot wounds constituted most of the remaining penetrating trauma patients (142 of 144 patients: 97%) (6).

Temporary intravascular shunting is indicated in (1) open extremity fractures with extensive soft tissue injury and concurrent arterial injury (Gustilo IIIC), (2) need for perfusion during complex vascular reconstruction, (3) damage control for patients in extremis, (4) perfusion prior to limb replantation, (5) truncal vascular control, and (6) complex repair of zone III neck injuries.

## Vascular Shunts in the Military

The use of TIVS has been well documented in the military literature. However, the initial concept of shunts was not that of a temporary conduit. Prior to 1900, blood vessel ligation was the standard repair strategy of vascular injury. During World Wars I and II, shunts were considered “inventions of necessity” as they offered the sole alternative to ligation which was equivalent to amputation in many cases. Shunts were intended to be permanent and were used to maintain limb perfusion while allowing for the development of collaterals that may maintain limb viability. The prolonged medical evacuation time ranging between 12 and 15 h precluded the limited surgical bypass interventions. Shunting as a temporary intervention was made possible with the reduction of evacuation times that in turn allowed the development of vascular reconstruction techniques. The Afghanistan and Iraq wars reinforced interest in temporary vascular shunts with reports describing their effective use at forward facilities that allowed for damage control pending evacuation and definitive treatment at higher levels of care (7, 8).

Most of the recent data on vascular trauma come from the Balad Vascular Registry (BVR). The registry identifies consecutive patients with vascular injuries sustained during the Iraq military campaigns. Injuries requiring an attempt at limb salvage were included while patients requiring an immediate amputation were excluded from the registry. The data from Iraq reveal that vascular injuries comprise 5–6% of battlefield injuries; 80% of which are in the upper or lower extremities (9–12). Shunt incidence rate was variable and ranged between 17 and 24% of extremity vascular injuries according to the BVR and was around 53% according to the Navy and Marine Corps Combat Trauma Registry (NMCCTR) that captures extremity injuries in Iraq and Afghanistan (9, 10). Femoropopliteal injuries from the BVR demonstrated high shunting rates between 38 and 45%. Shunt use also depended on the anatomic location of an injury (proximal vs. distal extremity), the nature of the vessel (artery vs. vein), casualty flow, and the casualty’s proximity to the next echelon of care. Injuries received at an Echelon II facility in Iraq had shunts placed in 5/7 (71.4%) proximal upper extremity injuries and 20/41 (48.7%) proximal lower extremity injuries (13). Definitive vascular repair was not recommended at these facilities given the inherent hostile environment and lack of medical resources. According to a query of the NMCCTR, 100% of shunts were placed at Echelon II facilities whereas all the injuries received at Echelon III facilities were treated with definitive repair (10).

Shunts have proven their superiority compared to ligation. Current amputation rates are well below the 72% rate for popliteal injuries and the 53% rate for femoral injuries in the Second World War; most of these injuries were treated with ligation (2). An analysis of amputation rates at 2 years of follow-up for vascular injuries in Iraq and Afghanistan revealed an overall amputation rate of 16% (10). The rate with femoropopliteal injuries from the BVR was around 7% (14). The reduction in current military amputation rates is attributable to many factors, such as the advancement of forward surgical facilities, rapidity of evacuation, and possibly the use of shunts as temporizing damage control adjuncts prior to definitive repair at another more equipped facility. Shunting has become the standard management in military traumas with several case series and retrospective studies demonstrating its success (10, 15, 16).

Proximally placed shunts have a superior patency compared to distal shunts given that distal arteries are more prone to vasospasm under shock conditions (9). Queries of the BVR showed that proximal vascular shunt patency rates ranged between 78 and 96% compared to 12 and 18% for distal shunts (9, 12–14, 17). Despite the difference in patency rates between shunts placed in the proximal and distal arteries, early limb viability rates were similar between the two groups which questions the role of distal shunts in limb salvage.

A case–control comparative study between TIVS use prior to repair vs. definitive primary repair alone was conducted between 2003 and 2007 with vascular trauma cases obtained from multiple theater trauma registries coming out of the military campaigns in Iraq. The groups were matched according to a patient’s age, location of injury (upper vs. lower extremity), and the need for a major vascular operation during the same time period. The study showed similar amputation rates between the two groups (TIVS: 13–19% vs. No TIVS: 21–23%; *P* > 0.05), although the mean time to amputation was longer for the TIVS compared to the control group (TIVS: 105 days vs. no TIVS: 15 days) (16). The authors then proposed a model looking at the predictors of limb salvage. Associated bone injury, elevated Mangled Extremity Severity Score, and venous ligation were found to be the predictors of amputation (*P* < 0.05). TIVS use was not among the predictors although there was a trend toward a lower amputation (relative risk = 0.47, *P* = 0.11) (16). The shunt group, however, had a higher Injury Severity Score and blood component requirements, suggesting that shunts might attenuate the systemic response to injury. Without shunting, that group might have ended up with inferior outcomes. The authors concluded that temporary shunting was not associated with worse outcomes while suggesting some benefit with shunt use although statistical significance was not reached.

Despite their prevalence in the military, shunts should not be viewed as risk free. Complication rates range between 0 and 4.7% and consist mainly of thrombosis and dislodgement during transport (10, 14, 16). Debakey expressed another concern with shunt use when he mentioned that “additional arterial substance may be destroyed in the course of attempting to insert the tube” (2, 7). This may have implications on the patency of the interposition grafts fashioned at the sites of shunt insertion.

## Vascular Shunts in Civilian Trauma

The civilian sector later caught up with the military experience albeit under different circumstances of shunt placement. The mechanisms of injury, indications and incidence of use are quite different and as such it is difficult to extrapolate data from the military. A recent analysis of the NTDB demonstrated that 74% of vascular shunts are placed in “blunt traumas associated with extensive orthopedic and/or soft tissue injury” compared to 1% in military registries (6, 16). The incidence of shunt use in civilian vascular trauma is also lower and ranges between 3 and 9% (3, 18). A query of the NTDB over a 5-year period revealed that only 6 centers used more than 5 shunts during this period; only 3 used more than 10 (6). The diverse nature of this population was highlighted when the NTDB revealed that 64% of vascular injuries were blunt traumas in contrast to earlier and recent studies indicating a 62–71% incidence of penetrating injuries (3, 18). This variability showcases the inherent selection bias with single center studies depending on the regional characteristics of an institution.

## Evidence in Favor of Shunt Use

The two most common indications for shunt use in civilian trauma are damage control and as a temporizing measure for orthopedic fixation. Trauma societal guidelines recommend minimizing the ischemic time to less than 6 h to allow for maximum limb salvage (19, 20). Restoration of blood flow through temporary shunting is suggested in the presence of a concomitant bone injury, while immediate vascular repair is advised for “stable skeletal injuries” (21).

The prevalence of shunt use in the military and the staggering decrease in amputation rates across decades have standardized shunt use. Data from the Military Liaison Committee revealed that only 4% of a civilian surgeon panel reported that “shunts rarely work” (22).

The high limb salvage and relatively low complication rates associated with shunts make them quite appealing. Damage control in the context of an iliac artery injury is typically limited to surgical ligation or temporary shunting; vascular reconstruction in the acute scenario is unlikely. Compared to ligation, TIVS reduced amputation, fasciotomy and mortality rates from 47 to 0%, 93 to 43%, and 73 to 43%, respectively, in patients with iliac artery injuries requiring damage control interventions (23). It is inevitable for shunting to yield superior outcomes compared to ligation. However, the true comparison (if possible) should be between shunting and vascular reconstruction. A retrospective review of 17 patients with blunt popliteal injuries, shunting (seven patients) was shown to reduce ischemia time, fasciotomy and amputation rates, and repeat operations compared to the non-shunted group (10 patients) (24).

The largest and most recent multicenter study revealed that shunts are infrequently used for vascular injuries with only 213 TIVS placed across 9 years (2.7% of vascular injuries) (18). Two-thirds of the shunts were placed for damage control with the remaining one-third for combined orthopedic-vascular injuries. Only once was shunt insertion due to lack of surgical expertise. The elevated mortality rate among shunted injuries in the study (20.4%) was due to truncal injuries constituting 25% of the study population; none of the deaths was attributed to shunt use. The amputation rate was 3.5% with half of them due to soft tissue injuries, while the other half was due to graft complications; none of the amputations was attributed to shunt use (18).

An earlier 10-year analysis from a Level I trauma center looking at 99 shunted vascular injuries showed a 9% shunt incidence rate. Damage control (44%) and orthopedic-vascular injuries (42%) were the most frequent shunt indications. The mortality rate (12.0%) was lower compared to the previous multicenter study probably due to the lower rate of truncal vascular injuries (3). They had a higher amputation rate of 16.6% which could be attributed to the higher proportion of popliteal injuries (31.8 vs. 18.8%) (3).

Shunts were more commonly used in extremity (65–94%) and arterial (70–100%) injuries compared to truncal and venous injuries, respectively (3, 18). The most commonly shunted extremity vessels were the superficial femoral artery (25%) followed by the popliteal artery (19%) (3, 18). Although no differences among shunt types have been reported, the Argyle shunt (C.R. Bard, Billerica, MA, USA) followed by the Pruitt-Inahara shunts (LeMaitre Vascular, Burlington, MA, USA) have been the most commonly utilized conduits (3, 18). Chest tubes have been used for larger vessels with the majority being for truncal aortic injuries (18). Nasogastric tubes were used to fashion shunts in one study from South Africa (25). There was no association between shunt thrombosis and the use of non-commercial shunts (chest tube/feeding tube) (18). However, non-commercial shunts and “damage control” shunt indication were associated with higher odds of subsequent graft failure (OR = 6.2, OR = 3.3, *P* < 0.05, respectively).

Venous injury adds more treatment heterogeneity to vascular trauma. One study reported ligating all venous injuries (25). There is evidence, however, that vein shunting and repair is associated with lower incidences of compartment syndromes, fasciotomies and amputations (26, 27). Shunt diameter is another critical consideration; oversizing causes intimal injury while undersizing might cause shunt dislodgement. Shunt dwell time has not been associated with thrombosis; 86.5% of shunts were removed at 24 h in one study, while the mean “dwell” time was 24 h in another (3, 18). Current shunt configuration is in a straight position (vs. looped) inserted to a depth of 2 cm into the injured vessel (8, 26).

There is no consensus surrounding the factors associated with shunt thrombosis although shunt sizing and vessel caliber have been implicated. One series demonstrated a shunt thrombosis rate of 5% all occurring in small caliber vessels (superior mesenteric and brachial arteries) (3). This is relevant since the majority of shunts (78%) are placed in larger caliber vessels (18).

## Evidence Against Shunt Use

The heterogeneity of vascular traumas in terms of injury location (truncal vs. extremity; proximal vs. distal) and severity, time to presentation, shunt type, trauma facility, surgeon expertise, and availability of resources makes the decision of shunting a difficult one. Given the multitude of factors involved with vascular trauma, the role shunts have in determining limb salvage and mortality appears minimal.

**Figure 1:**
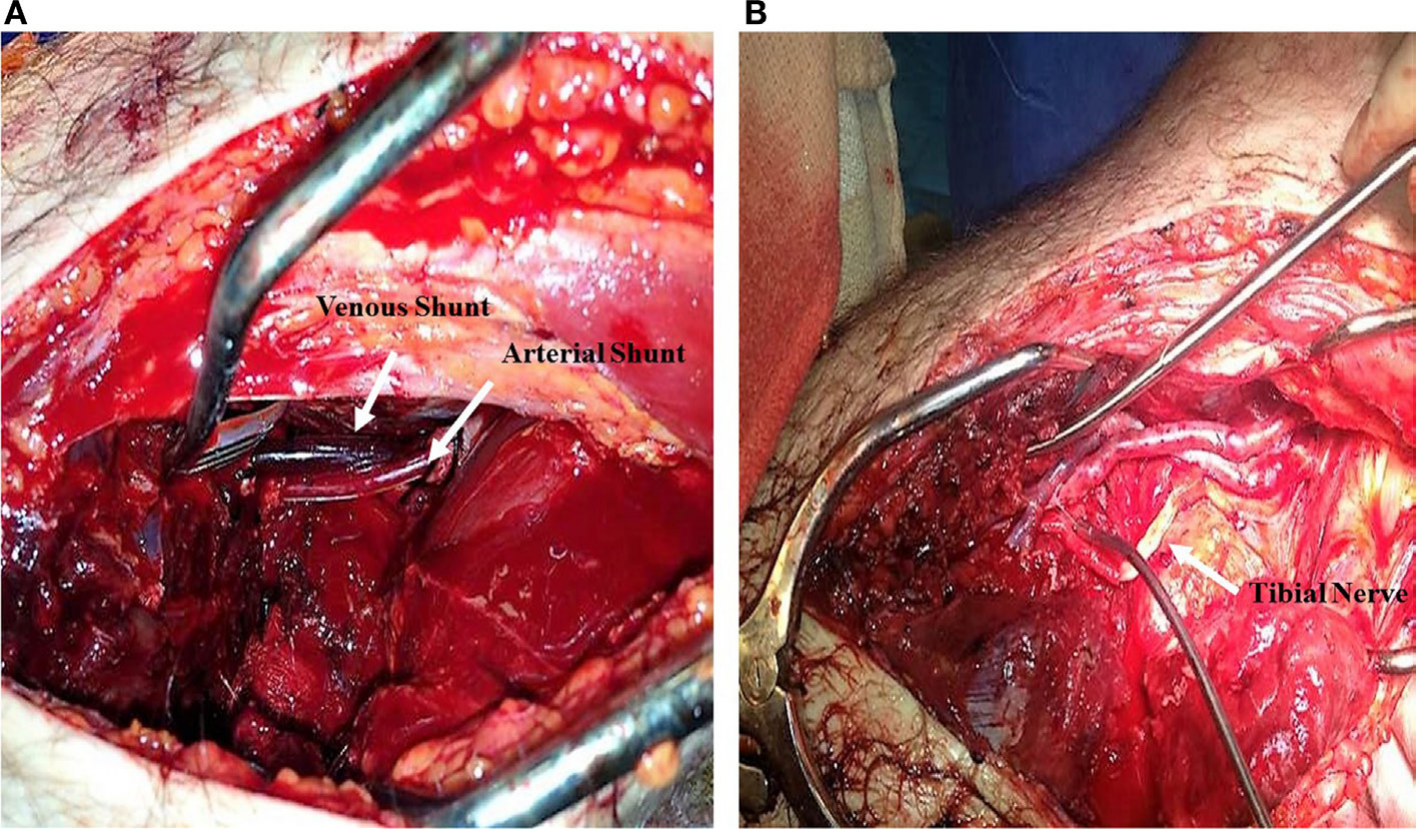
Popliteal artery and vein transection secondary to a motor vehicle collision. **(A)** 12 and 14 French Argyle shunts placed in the popliteal artery and vein, respectively. **(B)** Vascular reconstruction of tibial artery and vein using a Great Saphenous Vein interposition graft; Tibial nerve reconstructed with artificial synthetic tubulization.

**Figure 2:**
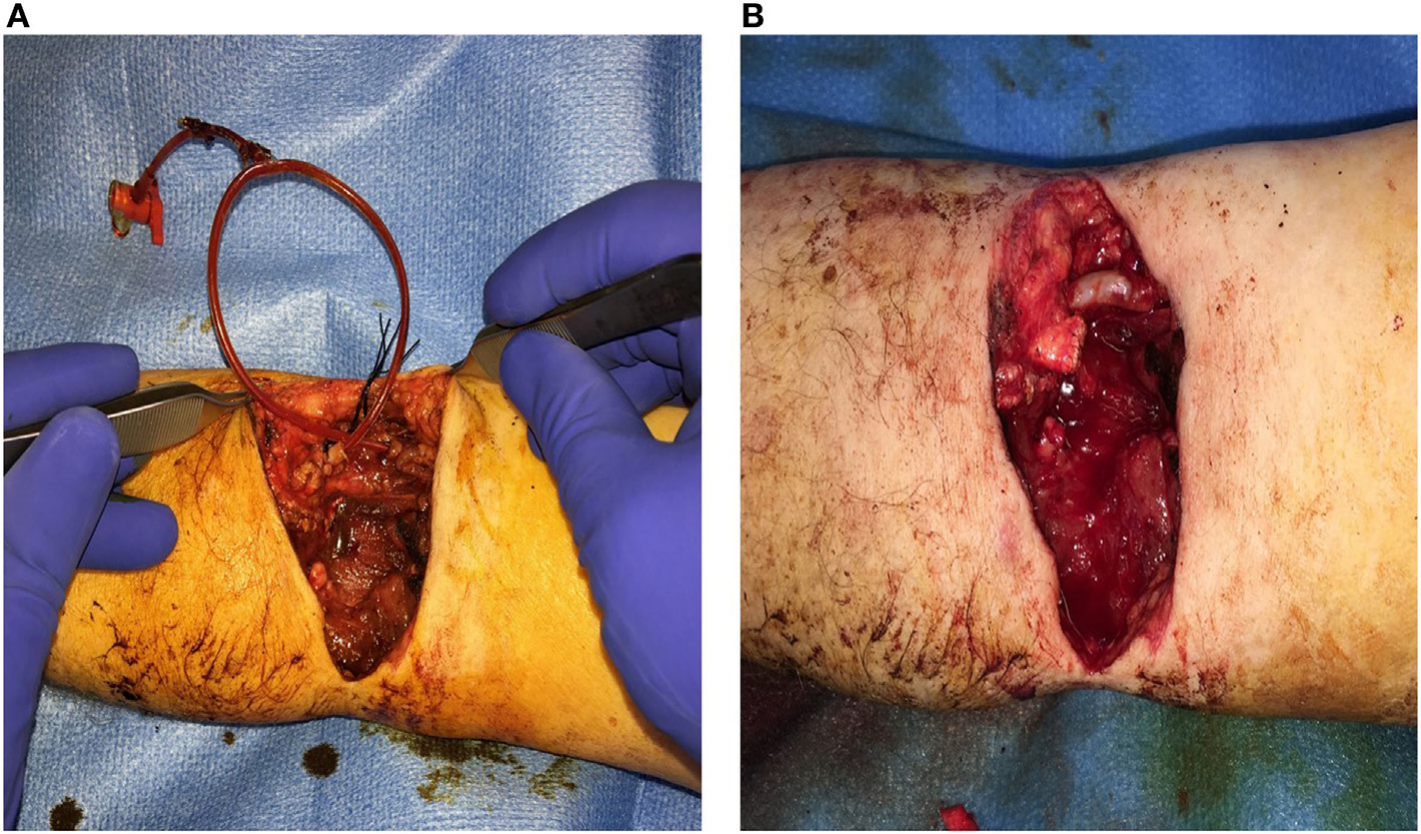
Brachial artery transection secondary to an electric saw injury. **(A)** 12 French Pruitt-Inahara Shunt placed in the Left Brachial artery. **(B)** Vascular reconstruction of artery using a Great Saphenous Vein interposition graft.

While the aforementioned shunting series portray favorable outcomes, the absence of a control group (primary or definitive vascular repair without prior vascular shunting) is a major limitation in most studies describing vascular shunt use. While it is true that compared to ligation shunting has superior outcomes, the same cannot be extrapolated when we compare shunts to definitive surgical repair (16, 23). One would assume that shunts would be most needed in small, rural centers with limited access to vascular surgeons and appropriate resources yet most of the literature supporting shunts comes from Level I trauma centers that are well-equipped and capable of definitive vascular surgical repair. However, the literature and our own experience as a Level I trauma center support the notion that shunts are not that common in modern-day vascular trauma. In our series of 149 patients with extremity injuries over a 10-year period, 2% only required a shunt (Figures 1 and 2) (28). The mechanism of injury (blunt vs. penetrating), presence of multiple tibial injuries, a popliteal injury, and a pulse deficit were significant predictors of delayed amputation; the numbers were insufficient to assess the role of shunts in determining limb outcomes.

Popliteal injuries have been historically and presently associated with the greatest risk of limb loss from a peripheral vascular injury (2, 29). A recent NTDB query (1989–2003) of popliteal injuries revealed that the amputation rates with blunt and penetrating injuries were 18 and 9%, respectively (29). While this database lacks information about shunting, the main predictors of amputation were associated bony fractures and soft tissue and nerve injuries. The more recent experience (1997–2007) of combined shunting of the popliteal artery and vein demonstrated 40 and 22% amputation rates for blunt and penetrating traumas, respectively (3). Therefore, shunting may play a role in preserving early limb viability, however, its role in limb salvage appears modest in the presence of other complicating factors.

The South African experience with civilian vascular trauma from a single level I trauma center revealed that shunt complication rates can reach up to 20%. In that study, three out of the seven complications were shunt dislodgements or migrations (one of which ended in death) with the other four being shunt thromboses (25). The most recent multicenter civilian trauma study illustrated a 5.6% shunt thrombosis and 1.4% shunt dislodgement rates (18). Most studies indicate that shunts were not responsible for the amputations and attribute limb loss soft tissue damage sustained at the time of the trauma. However, shunts have been associated with endothelial injury and subsequent vascular graft thrombosis. One study attributed half of the amputations to “tissue ischemia from graft failure” (18).

Data from iliac vessel injuries demonstrate a high mortality rate. Shunt use with these injuries was associated with a 33% (2/6) mortality rate compared to 24% (15/63) in patients that did not receive a shunt; shunting did not appear to affect the mortality rates in this population (30). An earlier study of iliac vessel injuries that did not use TIVS reported a mortality rate of 25% in patients with isolated arterial injury treated with a bypass (31). Another potential disadvantage with shunt use is the inability to perform primary vessel repair. One study reports that only 10 of 23 arterial injuries were primarily repaired attributing this to possible vessel injury by the shunt and the need for debridement (30).

Single center studies carry major selection biases that preclude generalizable data. While registries circumvent this limitation, they lack the required level of detail and follow-up to come up with meaningful conclusions. Comparisons between shunting and surgical ligation will inevitably favor shunt use; retrospective reviews of shunting at level I trauma centers will also yield favorable results given the advanced surgical expertise and technological development. Randomized controlled clinical trials offer high-quality evidence, yet they are impractical given the low incidence of vascular traumas and the uneven distribution of vascular expertise amongst centers and physicians.

## Conclusion

Temporary intravascular shunt placement in civilian vascular injury is uncommon and still operators nationwide may be hesitant in using it more widely. Given the inherent nature of extremity injury, its complexity, and need for urgent decisions, it is unlikely that a prospective good quality comparative study will ever be designed. It is, however, reasonable to consider a temporary proximal vascular shunt for the patient in the extremis for damage control or as a temporizing measure for orthopedic fixation. Shunting in damage control situations is per physician preference. Multiple factors come into play particularly the patient’s age, hemodynamic stability, and need for pressors. The decision takes into consideration input from members of the managing team (vascular, trauma, orthopedic, Intensive Care Unit, and anesthesia). As such, there is no generalized approach; it is rather patient and clinical scenario specific.

## Author Contributions

AA and EA: conception and design, analysis and interpretation, writing of the article, critical revision, and final approval of manuscript. KS: analysis and interpretation, writing of the article, critical revision, and final approval of manuscript. LA, GB, EP, and RC: analysis and interpretation, critical revision, and final approval of manuscript.

## Conflict of Interest Statement

The authors declare that the research was conducted in the absence of any commercial or financial relationships that could be construed as a potential conflict of interest.

## References

[1] Moore WS Vascular and Endovascular Surgery: A Comprehensive Review. 8th ed Philadelphia: Elsevier Inc (2013). 1112 p.

[2] Debakey MEE Simeone FA Battle injuries of the arteries in World War II; an analysis of 2,471 cases. Ann Surg (1946) 123(4):534–79. 10.1097/00000658-194604000-00005 PMC1803573 17858758

[3] Subramanian A Vercruysse G Dente C Wyrzykowski A King E Feliciano DV A decade’s experience with temporary intravascular shunts at a civilian level I trauma center. J Trauma (2008) 65(2):316–24; discussion 324–6. 10.1097/TA.0b013e31817e5132 18695465

[4] Tan T-W Joglar FL Hamburg NM Eberhardt RT Shaw PM Rybin D Limb outcome and mortality in lower and upper extremity arterial injury: a comparison using the National Trauma Data Bank. Vasc Endovascular Surg (2011) 45(7):592–7. 10.1177/1538574411415125 21984027

[5] Eger M Golcman L Goldstein A Hirsch M The use of a temporary shunt in the management of arterial vascular injuries. Surg Gynecol Obstet (1971) 132(1):67–70. 5538814

[6] Ball CG Kirkpatrick AW Rajani RR Wyrzykowski AD Dente CJ Vercruysse GA Temporary intravascular shunts: when are we really using them according to the NTDB? Am Surg (2009) 75(7):605–7. 19655605

[7] Hancock CH Rasmussen TE Walker AJ Rich NM History of temporary intravascular shunts in the management of vascular injury. J Vasc Surg (2010) 52(5):1405–9. 10.1016/j.jvs.2010.04.060 20615647

[8] Ding W Wu X Li J. Temporary intravascular shunts used as a damage control surgery adjunct in complex vascular injury: collective review. Injury (2008) 39(9):970–7. 10.1016/j.injury.2008.01.008 18407275

[9] Rasmussen TE Clouse WD Jenkins DH Peck MA Eliason JL Smith DL The use of temporary vascular shunts as a damage control adjunct in the management of wartime vascular injury. J Trauma (2006) 61(1):8–15. 10.1097/01.ta.0000220668.84405.17 16832244

[10] Borut LJ Acosta CJA Tadlock LM Dye JL Galarneau M Elshire CD. The use of temporary vascular shunts in military extremity wounds: a preliminary outcome analysis with 2-year follow-up. J Trauma (2010) 69(1):174–8. 10.1097/TA.0b013e3181e03e71 20622589

[11] Rasmussen TE Clouse WD Jenkins DH Peck MA Eliason JL Smith DL. Echelons of care and the management of wartime vascular injury: a report from the 332nd EMDG/Air Force Theater Hospital, Balad Air Base, Iraq. Perspect Vasc Surg Endovasc Ther (2006) 18(2):91–9. 10.1177/1531003506293374 17060224

[12] Clouse WD Rasmussen TE Peck MA Eliason JL Cox MW Bowser AN In-theater management of vascular injury: 2 years of the Balad Vascular Registry. J Am Coll Surg (2007) 204(4):625–32. 10.1016/j.jamcollsurg.2007.01.040 17382222

[13] Taller J Kamdar JP Greene JA Morgan RA Blankenship CL Dabrowski P Temporary vascular shunts as initial treatment of proximal extremity vascular injuries during combat operations: the new standard of care at echelon II facilities? J Trauma (2008) 65(3):595–603. 10.1097/TA.0b013e31818234aa 18784573

[14] Woodward EB Clouse WD Eliason JL Peck MA Bowser AN Cox MW Penetrating femoropopliteal injury during modern warfare: experience of the Balad Vascular Registry. J Vasc Surg (2008) 47(6):1259–65. 10.1016/j.jvs.2008.01.052 18407450

[15] Granchi T Schmittling Z Vasquez J Schreiber M Wall M. Prolonged use of intraluminal arterial shunts without systemic anticoagulation. Am J Surg (2000) 180(6):493–7. 10.1016/S0002-9610(00)00508-0 11182405

[16] Gifford SM Aidinian G Clouse WD Fox CJ Porras CA Jones WT Effect of temporary shunting on extremity vascular injury: an outcome analysis from the Global War on Terror vascular injury initiative. J Vasc Surg (2009) 50(3):549–56. 10.1016/j.jvs.2009.03.051 19595542

[17] Chambers LW Green DJ Sample K Gillingham BL Rhee P Brown C Tactical surgical intervention with temporary shunting of peripheral vascular trauma sustained during Operation Iraqi Freedom: one unit’s experience. J Trauma (2006) 61(4):824–30. 10.1097/01.ta.0000197066.74451.f3 17033547

[18] Inaba K Aksoy H Seamon MJ Marks JA Duchesne J Schroll R Multicenter evaluation of temporary intravascular shunt use in vascular trauma. J Trauma Acute Care Surg (2016) 80(3):359–65. 10.1097/TA.0000000000000949 26713968

[19] Fox N Rajani RR Bokhari F Chiu WC Kerwin A Seamon MJ Evaluation and management of penetrating lower extremity arterial trauma: an Eastern Association for the Surgery of Trauma practice management guideline. J Trauma Acute Care Surg (2012) 73(5 Suppl 4):S315–20. 10.1097/TA.0b013e31827018e4 23114487

[20] Feliciano DV Moore EE West MA Moore FA Davis JW Cocanour CS Western Trauma Association critical decisions in trauma: evaluation and management of peripheral vascular injury, part II. J Trauma Acute Care Surg (2013) 75(3):391–7. 10.1097/TA.0b013e3182994b48 23928739

[21] Reber PU Patel AG Sapio NL Ris HB Beck M Kniemeyer HW. Selective use of temporary intravascular shunts in coincident vascular and orthopedic upper and lower limb trauma. J Trauma (1999) 47(1):72–6. 10.1097/00005373-199907000-00017 10421190

[22] Rasmussen TE Dubose JJ Asensio JA Feliciano DV Fox CJ Nunez TC Tourniquets, vascular shunts, and endovascular technologies: esoteric or essential? A report from the 2011 AAST Military Liaison Panel. J Trauma Acute Care Surg (2012) 73(1):282–5. 10.1097/TA.0b013e3182569df4 22743396

[23] Ball CG Feliciano DV. Damage control techniques for common and external iliac artery injuries: have temporary intravascular shunts replaced the need for ligation? J Trauma (2010) 68(5):1117–20. 10.1097/TA.0b013e3181d865c0 20453767

[24] Hossny A. Blunt popliteal artery injury with complete lower limb ischemia: is routine use of temporary intraluminal arterial shunt justified? J Vasc Surg (2004) 40(1):61–6. 10.1016/j.jvs.2004.03.003 15218463

[25] Oliver JC Gill H Nicol AJ Edu S Navsaria PH. Temporary vascular shunting in vascular trauma: a 10-year review from a civilian trauma centre. S Afr J Surg (2013) 51(1):6–10. 10.7196/sajs.1504 23472645

[26] Hornez E Boddaert G Ngabou UD Aguir S Baudoin Y Mocellin N Temporary vascular shunt for damage control of extremity vascular injury: a toolbox for trauma surgeons. J Visc Surg (2015) 152(6):363–8. 10.1016/j.jviscsurg.2015.09.005 26456452

[27] Barros D’Sa AAB Harkin DW Blair PHB Hood JM McIlrath E. The belfast approach to managing complex lower limb vascular injuries. Eur J Vasc Endovasc Surg (2006) 32(3):246–56. 10.1016/j.ejvs.2006.02.004 16618547

[28] Liang NL Alarcon LH Jeyabalan G Avgerinos ED Makaroun MS Chaer RA. Contemporary outcomes of civilian lower extremity arterial trauma. J Vasc Surg (2016) 64(3):731–6. 10.1016/j.jvs.2016.04.052 27444360 PMC5002387

[29] Mullenix PS Steele SR Andersen CA Starnes BW Salim A Martin MJ. Limb salvage and outcomes among patients with traumatic popliteal vascular injury: an analysis of the National Trauma Data Bank. J Vasc Surg (2006) 44(1):94–100. 10.1016/j.jvs.2006.02.052 16828431

[30] Oliver JC Bekker W Edu S Nicol AJ Navsaria PH. A ten year review of civilian iliac vessel injuries from a single trauma centre. Eur J Vasc Endovasc Surg (2012) 44(2):199–202. 10.1016/j.ejvs.2012.05.012 22658775

[31] Haan J Rodriguez A Chiu W Boswell S Scott J Scalea T. Operative management and outcome of iliac vessel injury: a ten-year experience. Am Surg (2003) 69(7):581–6. 12889621

